# Systematic analysis of DNA damage induction and DNA repair pathway activation by continuous wave visible light laser micro-irradiation

**DOI:** 10.3934/genet.2017.1.47

**Published:** 2017-02-21

**Authors:** Britta Muster, Alexander Rapp, M. Cristina Cardoso

**Affiliations:** Cell Biology and Epigenetics, Department of Biology, Technische Universität Darmstadt, 64287 Darmstadt, Germany

**Keywords:** DNA repair, DNA damage, processive DNA synthesis, laser micro-irradiation, live-cell microscopy

## Abstract

Laser micro-irradiation can be used to induce DNA damage with high spatial and temporal resolution, representing a powerful tool to analyze DNA repair *in vivo* in the context of chromatin. However, most lasers induce a mixture of DNA damage leading to the activation of multiple DNA repair pathways and making it impossible to study individual repair processes. Hence, we aimed to establish and validate micro-irradiation conditions together with inhibition of several key proteins to discriminate different types of DNA damage and repair pathways using lasers commonly available in confocal microscopes. Using time-lapse analysis of cells expressing fluorescently tagged repair proteins and also validation of the DNA damage generated by micro-irradiation using several key damage markers, we show that irradiation with a 405 nm continuous wave laser lead to the activation of all repair pathways even in the absence of exogenous sensitization. In contrast, we found that irradiation with 488 nm laser lead to the selective activation of non-processive short-patch base excision and single strand break repair, which were further validated by PARP inhibition and metoxyamine treatment. We conclude that these low energy conditions discriminated against processive long-patch base excision repair, nucleotide excision repair as well as double strand break repair pathways.

## 1. Introduction

Cellular DNA is constantly exposed to endogenous as well as exogenous genotoxic factors, leading to base damage, photoproducts/adducts or DNA single (SSBs) and double strand breaks (DSBs). If left unrepaired, these lesions can lead to mutations and subsequently cancer and/or cell death. To cope with these different sorts of DNA damage, several DNA repair pathways have evolved. Subtle modifications of DNA, such as base modifications, are repaired via base excision repair (BER) [1], whereas the repair of helix-distorting DNA damage, like e.g., photoproducts, is accomplished via the nucleotide excision repair pathway (NER) (reviewed in [2]). DNA double strand breaks can be repaired by either homologous recombination (HR) or non-homologous end joining (NHEJ) pathways (reviewed in [3]).

To explore these distinct DNA repair processes, *in vitro* biochemistry or yeast and mammalian cell genetic analyses have been extensively used and revealed detailed insights into their molecular mechanisms. The shortcoming of the above-mentioned approaches is that DNA repair is not studied in its natural context, i.e., the chromatin inside living cells. One technique that promises to overcome these limitations is laser microbeam irradiation. With the use of a focused laser beam, it is possible to induce DNA damage with a very high spatial resolution and tight temporal control inside of living cells [4]. This method can in principle be applied to a large variety of cells to study the kinetics of DNA repair *in situ* in the context of chromatin. Another great advantage is that with the use of genetically encoded fluorescently-tagged versions of different DNA repair factors it is possible to study their association/dissociation at sites of induced DNA damage in real time with high temporal resolution (reviewed in [5]–[8]).

The first applications of micro-irradiation were performed with UVC laser light (257 nm) and showed a localized induction of DNA photoproducts [9]. Subsequently, laser micro-irradiation was also successfully employed within the UVA range (∼360 nm). For induction of DNA double strand breaks, cells were often pre-sensitized with halogenated thymidine analogs (BrdU, IdU), DNA dyes (Hoechst) or both [10]–[13], while the micro-irradiation with UVA lasers without sensitization was found to induce predominantly base damage and an aberrant DNA double strand break response [14]. However, these sensitizers potentially induce DNA damage by themselves and have considerable effects on different aspects of cellular physiology, e.g., the cell cycle [15] or viability. To bypass this problem, micro-irradiation was also performed without sensitizers and it was demonstrated that micro-irradiation alone (365 nm) leads to DNA damage, including DNA double strand breaks [16]. Nevertheless, using this technique to image the earliest time points in the range of seconds or even milliseconds directly after DNA damage, requires coupling the UVA laser into a (confocal) microscope [11],[16] with specialized UV optics. In contrast, continuous wave (CW) 405 nm lasers are nowadays available in most confocal microscope systems and are compatible with standard optics. This wavelength was also shown to induce DNA damage after pre-sensitization of the cells, leading to the activation of different DNA repair pathways [15],[17]–[21].

However, as with UVA lasers, 405 nm laser micro-irradiation conditions also lead to the induction of several other types of DNA damage, which result in the activation of various DNA repair pathways [15],[22] and makes it impossible to study individual DNA repair pathways separately. To overcome this challenge, other lasers were tested. It was shown that with multi-photon laser systems such as a pulsed 1050 nm lasers it is possible to favor the induction of DSBs over cyclobutane pyrimidine dimers (CPD) when compared with 775 nm pulsed lasers [22]–[24]. Furthermore, micro-irradiation with 266 nm laser predominantly formed typical UV photo-lesions such as CPDs and 6-4 photoproducts (6-4PP) and only induced DSBs after high intensity irradiation [15]. Nevertheless it was also shown that with pulsed lasers not only DSBs are induced, but that these DSBs have a chemically distinct structure, namely a more complex structure, compared to ultra-soft X-rays or even enzymatically induced DSBs [25]. This complexity affects the resulting DNA repair kinetics. Taken together these studies show that it is possible to discriminate or at least enrich strongly for specific types of DNA damage by selecting appropriate wavelength and power settings. Yet, these discriminations are based on non-linear multi-photon effects and/or require special lasers together with special lenses (UV transmitting) not commonly available in most laboratories [26],[27], such as a frequency doubled, pulsed Nd:YAG laser to induce high densities of DNA damage [22],[28].

Consequently, our aim was to achieve discrimination for specific types of DNA lesions and repair pathways with lasers commonly available on most confocal microscopes. We tested four different wavelengths (405, 488, 561 and 633 nm) with and without sensitizers on a spinning disk confocal microscope as well as on a laser scanning confocal microscope with laser energies ranging from 1 to 66 mJoule (mJ) in the objective plane. After validation of the type of DNA damage induced using different DNA damage markers, we analyzed the activation of the different DNA repair pathways with the help of a set of fluorescently tagged DNA repair factors specific to the different repair pathways. We were able to establish conditions to discriminate for the non-processive short-patch base excision repair/SSB repair over processive DNA repair pathways using the 488 nm and partly also the 561 nm lasers, respectively.

## 2. Materials and Methods

### 2.1. Cell culture and transfection

HeLa Kyoto [29] cells, PARP^−/−^ MEFs [30], wild type 3T3 MEFs and GM00637 wild type human fibroblasts [31] were grown at 37 °C 5% CO_2_ in DMEM supplemented with 10 % FCS and 1 µg/mL gentamycin. HeLa Kyoto cells stably expressing mCherry-PCNA [32] were cultivated in the presence of blasticidin (2.5 μg/mL). All cell lines used have been provided and described in detail as indicated in the references quoted.

Cells grown on cover slide dishes (Glass Bottom Dishes, MatTek, 1.5 coverglass thickness) or in 8-well chambered coverslips (Nunc, Lab-Tek, Chambered Coverglass) were transfected with mammalian expression constructs coding for fluorescently tagged proteins using polyethylenimine as previously described [33]. For discrimination of different DNA repair pathways the following fluorescently tagged proteins were used: mCherry-PCNA [34],[35] (pc1322), GFP-DNA Ligase 4 [18] (pc1142), Ku70-GFP [36] (pc1185), XPC-GFP [37] (pc1328), XPG-GFP [28] (pc1329), mRFP-XPA [38] (pc1393), GFP-DNA Ligase 3 [18],[19] (pc1139), GFP-p66 (subunit of DNA polymerase delta) [39] (pc1276), GFP-Fen1 [35] (pc1112), NBS1-GFP [40] (pc1784), GFP-RPA-70 (pc502), GFP-OGG1 (pc2369) (kind gift from U. Müller and H. Leonhardt, LMU, Munich), pGFP-polb (pc2455).

Human RPA-70 was subcloned from p11d-RPA70 [41] as a BstBI/XmaI fragment into the ClaI/NgoMI sites of the pEhNt vector to generate a GFP-RPA-70 fusion.

Human DNA polymerase beta was subcloned from pIRESpuro-Flag-Pol b [42] into pEGFP-C3 (Clontech) using the NotI, PspOMI sites to generate a GFP-Pol b fusion.

GFP-Rad51 (pc2449), GFP-XRCC1 (pc1152), mRFP-XRCC1 (pc1156), GFP-APEX (pc2365) and mRFP-APEX (pc2366) constructs were generated by cloning the corresponding human cDNA into either pEGFP-C1 or pmRFP-C1 backbone (Clontech) vectors. The following primers were used for PCR amplification (restriction sites used for cloning are in bold): XRCC1 forward 5′ AA **ACCGGT** ATGCCGGAGATCCGCCTCC 3′ (**HpaI)**, XRCC1 reverse 5′ AA **GCTAGC** GGCTTGCGGCACCACCCC 3′ (**NheI**), APEX1 forward: 5′ AA **ACCGGT** ATGCCGAAGCGTGGGAAAAAGG 3′ (**HpaI**), APEX1 reverse: 5′ AA **GCTAGC** CAGTGCTAGGTATAGGGTGATAGG 3′ (**NheI**), Rad51 forward 5′ AA **GTCGAC**GTAATGGCAATGCAGATGC 3′ (**SalI**), Rad51 reverse: 5′ AA **GGATCC**AAGTCTTTGGCATCTCCCACTC 3′ (**BamHI**).

All constructs were verified by sequencing.

For cell cycle staging using the fluorescent ubiquitination-based cell cycle indicator system, we used the plasmids pFucci-G1 Orange and pFucci-S/G2-M Green (Amalgaam MBL) [43]. For inhibition of PARP, cells were pretreated for one hour under standard cell culture conditions in the presence of 10 µM Olaparib (Axon Medchem BV). Also micro-irradiation was performed in the presence of the inhibitor.

To block abasic sites with methoxyamine (MX, Sigma Aldrich), cells were pre-incubated for one hour under standard cell culture conditions in the presence of 6 mM MX. Micro-irradiation was also performed in the presence of MX.

As additional exogenous sensitizers for low energy irradiation, either the thymidine analog bromodeoxyuridine (BrdU) or the DNA intercalating agent ethidium bromide (EthBr) were used. BrdU was added at a final concentration of 10 µM over night before the irradiation was carried out and EthBr was added at a final concentration of 200 nM 45 minutes before micro-irradiation.

### 2.2. Microscopy and micro-irradiation

Imaging and micro-irradiation experiments were performed using an UltraVIEW VoX spinning disc confocal system (Perkin Elmer) equipped with a FRAP unit (Perkin Elmer) in a closed live-cell microscopy chamber (ACU, Perkin Elmer) at 37 °C with 5% CO_2_ and 60% humidity, mounted on a Nikon TI microscope (Nikon). Images were taken with a CFI Apochromat 60x/1.45 NA oil immersion objective. GFP and cherry or mRFP were imaged with 488 and 561 nm laser excitation and 527 ± 55 and 612 ± 70 nm (full width at half maximum) emission filters, respectively.

For standard micro-irradiation, a preselected spot (∼1 µm diameter) within the nucleus was micro-irradiated for 1200 milliseconds with one of the following laser lines 405, 488, 561 or 633 nm laser set to 100% (Acousto Optic Tunable Filter, AOTF), resulting in 1, 3, 8 and 5 mJ respectively. Energy output of the different lasers was measured with a laser power meter (Ophir Optronics Solutions) directly after the objective with beam park settings. To assess the power density of the micro-irradiation condition in the focal plane, we used fluorescent slides (Diagnostic Slides #92001, Chroma Technology, for the 405 and 488 nm lasers) or dried fluorescent dye films (for the 561 nm laser) to measure the spot size of the micro-irradiation. For the spinning disk system the spot sizes were 1.8 ± 1.0 µm^2^ (405 nm), 2.9 ± 0.6 µm^2^ (488 nm) and 3.8 ± 1.2 µm^2^ (561 nm) while for the point scanning confocal the measured spot sizes were 4.2 ± 0.6 µm^2^, 2.8 ± 0.7 µm^2^ and 2.5 ± 1.3 µm^2^, respectively. This results in power densities of 47 (405 nm), 86 (488 nm) and 175 kW/cm^2^ (561 nm) for the spinning disk and 20 (405 nm), 89 (488 nm) and 266 kW/cm^2^ (561 nm) for the point scanning microscope, respectively. In terms of exposure these energy densities can be expressed as 55, 103 and 201 kJ/cm^2^ for the spinning disk microscope and 24, 107 and 320 kJ/cm^2^ for the point scanning confocal, respectively. Before and after micro-irradiation, confocal image series of one mid nucleus z section were recorded in 2–15 seconds intervals. For evaluation of the accumulation kinetics between four and 20 cells were analyzed. Images were first corrected for cell movement (ImageJ plug in StackReg, transformation mode: Rigid body) and mean intensity of the irradiated region was divided by mean intensity of the whole nucleus (both corrected for background) using ImageJ software. Maximal accumulation represents the highest ratio from each experiment. Maximal accumulation values were generally higher in the presence of exogenous sensitizers (see Table S1) and by selecting lower expressing cells. In the latter, the amount of accumulated protein at the irradiation spot is similar but the unbound nucleoplasmic species is lower, thus leading to a higher dynamic range of the measurements. Care was taken to ensure equal expression levels in comparative studies.

For validation on another microscope system, a Leica SP5 II confocal laser point scanning microscope was used. This microscope was equipped with a different set of lasers: a 405 diode laser, a 488 multi line Ar ion laser, a 561 nm DPSS laser and a 633 HeNe gas laser. All coupled into the microscope and controlled by an acousto-optical beam splitter (AOBS). For micro-irradiation experiments the HCX PL APO 63x/1.4 NA objective lens was used. Energy levels for each wavelength were adjusted to be equivalent to the experiments on the spinning disk microscope using the beam park settings of the Leica LAF software.

DNA damage induction and activation of DNA repair pathways with lower and higher energies were additionally tested on the spinning disc confocal system. For lower energies micro-irradiation was done for 200 or 800 milliseconds resulting in 0.1 and 0.6 mJ for 405 nm laser, 0.5 and 2 mJ for 488 nm laser and 1 and 5 mJ for 561 nm laser. For higher energy levels micro-irradiation with the 488 nm laser was done for 6.8 seconds resulting in 17 mJ and with the 561 nm laser for 10 seconds resulting in 66 mJ.

Half-times for XRCC1 accumulation were calculated from time of bleach till maximal accumulation using GraphPad Prism 5 one phase association (single exponential function: Y = Y_0_ + (Plateau-Y_0_) × (1−exp(−K × x))).

### 2.3. Immunofluorescence staining with DNA damage markers

HeLa Kyoto cells were micro-irradiated with the different wavelengths as described above, directly fixed after irradiation (maximum 15 min delay) and stained for different DNA damage markers or marker proteins. DAPI was used for DNA counterstaining. Unless otherwise noted, incubations were at room temperature.

For γH2AX and XRCC1 staining, HeLa Kyoto cells stably expressing mCherry-PCNA were used, to enable exclusion of S-phase cells and damage induction control. Cells were fixed with 3.7% formaldehyde for 15 min, permeabilized for 20 min in 0.5% Triton-X 100 in PBS, blocked for 30 min with 2% BSA/PBS and subsequently incubated with primary antibody mouse monoclonal anti-phospho-histone H2AX (Millipore, clone JBW301) diluted 1:200 or mouse monoclonal anti-XRCC1 (Abcam, clone 33-2-5) diluted 1:100 in 1% BSA/PBS for 1 h. Secondary antibodies (anti-mouse IgG Cy5, Jackson ImmunoResearch Laboratories, catalog 715-175-150) were diluted 1:400 in 1% BSA/PBS and also incubated for 1 h.

For detection of cyclobutane pyrimidine dimers (CPDs), cells were fixed for 10 min at −20 °C with ethanol and treated for 10 min on ice with 2 N HCl. After blocking for 30 min with 2% BSA/PBS, mouse anti-CPD antibody (Kamyia, clone KTM53) was diluted 1:200 in 1% BSA/PBS and incubated for 1 h. Secondary antibodies (anti-mouse IgG Alexa 488, Invitrogen, catalog A11029; 1:400 in 1% BSA/PBS) were also incubated for 1 h.

For TUNEL staining cells were fixed with 1% formaldehyde for 15 min, permeabilized 20 min with 0.5% Triton-X 100 and treated for 10 min at −20 °C with methanol/acetic acid (3:1). Terminal transferase reaction was performed according to the manufacturer's protocol (1x TdT buffer plus CoCl_2_ and 1 mM biotin-16-dUTP; New England BioLabs) plus additional ATP (1 mM) for 2 h at 37 °C. After blocking for 30 min with 2% BSA/PBS, Streptavidin-Alexa 488 (Invitrogen) was diluted 1:200 in 1% BSA/PBS and incubated for 30 min.

For PCNA staining cells were fixed with −20 °C methanol for 10 min, blocked for 30 min with 2% BSA/PBS and subsequently stained with mouse monoclonal anti-PCNA antibody (Dako, clone PC10) diluted 1:100 in 1% BSA/PBS and incubated for 1 h. Incubation with secondary anti-mouse IgG Cy5 (Jackson ImmunoResearch Laboratories, catalog 715-175-150, 1:400) was also performed for 1 h.

## 3. Results and Discussion

### 3.1. Micro-irradiation with 405 but not with 488 and 561 nm lasers induces DNA double strand breaks in the absence of exogenous sensitizers

**Figure 1.**
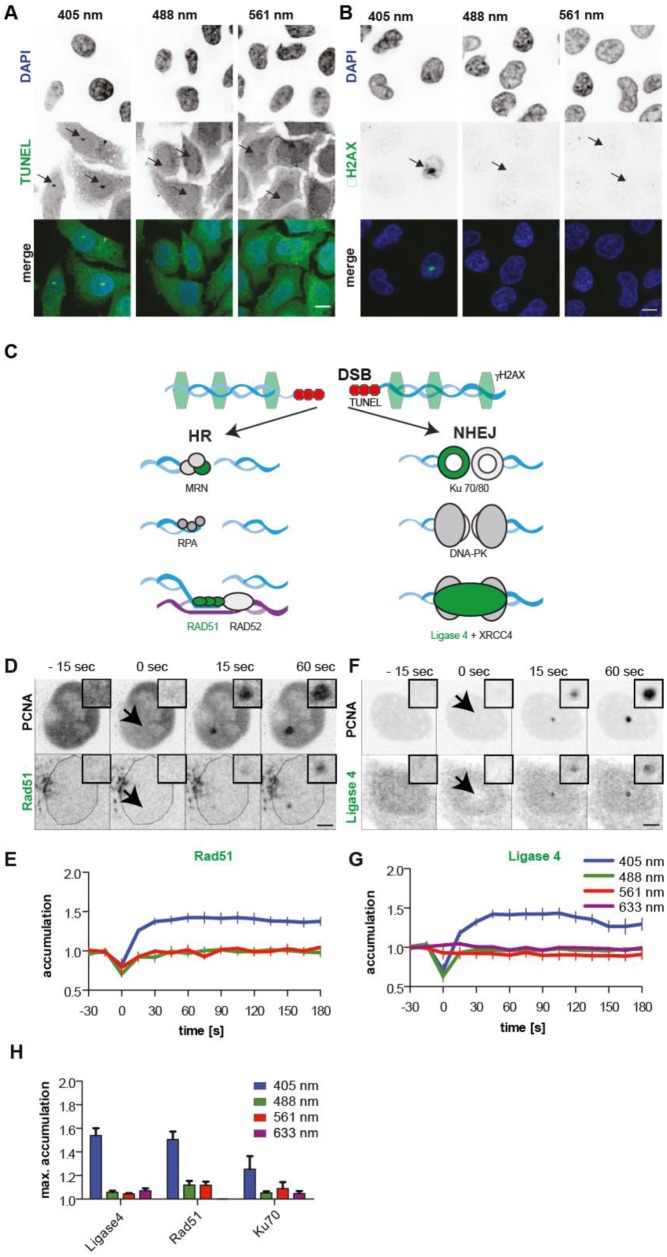
Laser micro-irradiation with 405 nm induces DSBs and activates homologous recombination and non-homologous end joining without exogenous sensitizers. HeLa Kyoto cells were micro-irradiated with 405, 488 or 561 nm laser lines at 1, 3, and 8 mJ respectively, fixed immediately (5 min lag time) and subsequently stained for the different DNA damage markers and DNA counterstained with DAPI. Arrows indicate the sites of micro-irradiation. Scale bars = 10 µm. Terminal deoxynucleotidyl transferase dUTP nick end labeling (TUNEL) (A) and immunofluorescence staining against the double strand break marker γH2AX (B) exclusively marks sites of 405 nm induced DNA damage and not after 488 or 561 nm. (C) Schematic representation of homologous recombination (HR) and non-homologous end joining (NHEJ), with analyzed proteins highlighted in green. (D) HeLa Kyoto cells, stably expressing mCherry-PCNA, were transfected with GFP-Rad51 (D) or GFP-Ligase 4 (F) and then irradiated with different laser lines as indicated at 1, 3, and 8 mJ for the 405, 488 and 561 nm lines respectively. Representative confocal microscopy images are shown from micro-irradiation experiments with 405 nm laser (D and F). Arrowheads point to the sites of micro-irradiation, shown enlarged as insets. Scale bars = 5 µm. (E and G) The plots show the accumulation of the different repair proteins over time as mean value. Error bars represent standard deviation. (H) Bar histograms display the calculated mean maximal accumulation for the different repair factors as indicated. Error bars represent standard deviation.

Micro-irradiation with 405 nm CW lasers without exogenous sensitizers was previously shown to induce a mixture of DNA damage, ranging from pyrimidine dimers to DNA double strand breaks [15]. DSBs induction was reported for higher laser energies [15],[17],[18],[44], or (pulsed) UVA lasers with BrdU sensitization [8],[45]. Dose estimates were made by comparing the local and global induction of RPA foci compared to heavy ion or X-ray irradiation. While the global damage induction was found to be equivalent to approximately 3 Gy, the local dose deposition was more in the range of hundreds of Gy for 0.3 MJ/m^2^ [8],[45]. This illustrates the difficulty of comparing local damage, as induced by laser micro-irradiation, to damage induced by ionizing radiation. To verify this type of damage, we used Terminal deoxynucleotidyl transferase dUTP Nick End Labeling (TUNEL) to tag open DNA ends. This method labels enzymatically free 3′OH DNA ends. A TUNEL positive signal was detected after micro-irradiation with the 405 nm laser, but not when cells were irradiated with longer wavelengths (Figure 1A). As an alternative marker for DSBs—the phosphorylated form of histone H2AX (γH2AX)—that appears within a few minutes after DSB induction [12] was used. Thus, we further validated the induction of DSBs using immunofluorescence with a γH2AX specific antibody. This marker was also exclusively detected after micro-irradiation with the 405 nm laser, but not with the 488 or 561 laser lines (Figure 1B). Although others reported the induction of DSBs also by 488 nm laser micro-irradiation, this only occurs at very high energy levels of 17 mJ [46], which is more than five times higher than the energy we applied. When we increased our 488 nm laser energy to this level, we were also able to detect the formation of γH2AX foci, indicating the formation of DSBs even with this longer wavelength (Figure S1A). Prompted by this observation, we also increased the energy level for the 561 nm laser up to 66 mJ. Again the formation of γH2AX foci was detectable (Figure S1A). Next, we performed an energy and wavelength dependency study to measure the detection threshold of γH2AX formation for the 405, 488 and 561 nm laser. When we analyzed the energy required for the detection of a significant γH2AX formation we could fit the data with both linear and quadratic regression with similar quality, although the 405 nm induced γH2AX phosphorylation showed a slightly better fit with a quadratic function (Figure S1B). While a linear dose dependency would suggest a replication dependent formation of DSBs, this possibility was excluded since S-phase cells (as ascertained by the subnuclear mCherry-PCNA pattern) were not used for micro-irradiation. In addition, as the time between irradiation and fixation was less than 15 minutes, it is very unlikely that the cells could enter S-phase and the replication machinery would be able to encounter the damage induced.

The formation of DSBs after micro-irradiation with visible light clearly shows that DNA can be damaged by these wavelengths. The mechanisms underlying how this damage is formed are still unclear. In principle DNA damage by irradiation either with UV or visible light can be accomplished by two different mechanisms. The first one is via direct absorption of a photon by the DNA. Since absorption of wavelengths over 300 nm directly by DNA is very low [47], this mechanism seems unlikely to account for all our observed DNA damage. Also two-photon effects, where photons with double the wavelength are simultaneously absorbed, are very unlikely considering the energy density used in these experimental conditions. The second mechanism is via absorption by endogenous, so far unknown, cellular photo-sensitizers. The so formed photo excited sensitizers can damage DNA either by a direct reaction with a DNA base (type I reaction) or via reaction with molecular oxygen (type II reaction). The latter leads to the formation of reactive oxygen species (ROS), which in turn reacts with DNA and forms oxidative damage like 8-oxoguanine or single strand breaks (SSB) [48]–[50]. When two SSB on opposing DNA strands are induced within a distance of less than 10–15 base pairs, these SSBs can convert into a DSB [22],[51]. Therefore, the observed DSBs are likely to be produced by multiple SSBs in close proximity. In a previous study, Tashiro et al. [13] estimated that the majority of damage induced by a 337 nm pulsed laser micro-beam are SSBs and oxidative damage. In un-sensitized cells only a minority of DSBs is expected (250–1000 times less compared to SSBs). Taking into account that we use even longer wavelengths and a CW 405 nm laser, we would expect an even higher ratio between SSBs and DSBs. Thus, DSBs are most likely generated from clustered direct SSBs or SSBs formed during BER [48]. To further validate this hypothesis, we analyzed the repair of induced DSBs in terms of RPA-70 focus resolution following 405 nm laser irradiation. Therefore, mCherry-PCNA and GFP-RPA-70 expressing cells were irradiated with 1 mJ of 405 nm laser and imaged for 16 hours (Figure S1C and D). The accumulation of RPA-70 is delayed as compared to PCNA accumulation. Additionally, it can be seen that the RPA-70 accumulation persists for the total time of 16 hours, whereas the PCNA repair focus disappears after about two hours. At about 6–7 hours, the cell progresses through the cell cycle and enters S-phase, as seen from the PCNA sub-nuclear replication pattern. The fact that RPA accumulation persists indicates that the DSBs induced by 405 nm lasers represent a difficult to repair structure, explainable with a high local clustering of SSBs, backbone and base modifications, in line with previous reports [22],[25],[45].

Taken together these data show that upon irradiation with moderate energy levels we only induce DSBs after micro-irradiation with 405 nm laser but not after micro-irradiation with 488 or 561 nm lasers. Only the application of very high energies can lead to the induction of these types of damage when 488 or 561 nm lasers are used.

### 3.2. Both DNA double strand break repair pathways NHEJ and HR are activated by 405 nm micro-irradiation

Since micro-irradiation with the 405 nm laser lead to the formation of γH2AX foci and a positive TUNEL staining, we tested whether the non-homologous end joining (NHEJ) and homologous recombination (HR) repair pathways were activated. The key proteins of each pathway are depicted in Figure 1C.

The activation of homologous recombination was monitored by GFP-tagged Rad51. Since overexpressed Rad51 protein forms higher-order nuclear structures even in the absence of DNA damage [52], we selected for our analysis only very low expressing cells. mCherry-tagged PCNA served as a reference for DNA damage induction, since PCNA has been shown to be recruited after micro-irradiation [34] and simultaneously allows cell cycle phase identification due to its specific subnuclear pattern during the S-phase. Micro-irradiation with the 405 nm laser at 1 mJ resulted in a strong (5 fold) (Figure 1D) recruitment of mCherry-PCNA and a significant accumulation of GFP-Rad51 (1.5 fold) to sites of DNA damage (Figure 1D and E). The addition of sensitizers (BrdU and EthBr) increased the accumulation of Rad51 following micro-irradiation with 405 nm laser at the same energy level to values of 2.2 and 3.6, respectively (Table S1). Micro-irradiation with the longer wavelengths on the other hand did not lead to a detectable accumulation of Rad51 at the sites of irradiation (Figure S2B and E), even in the presence of exogenous sensitizers (Table S1). Since DSB repair pathway activation is cell cycle dependent and HR is restricted to late S and G2 cells we analyzed the recruitment of Rad51 in a cell cycle dependent manner. For this we produced a cell cycle profile of the cell line during the micro-irradiation experiment based on the size of the nucleus (mid-nuclear section) of more than 100 cells. This resulted in a bimodal distribution (Figure S2A lower panel), from which we selected cells with a nuclear area <116 µm^2^ as G1 cells and cells with a nuclear area >360 µm^2^ as G2 cells. The size distribution was calibrated in an additional experiment to the cell cycle using the Fucci system. High levels of S-G2-M-green Fucci correlated with larger nuclear areas, while the G1-orange component dominated in smaller nuclei. As previously shown, Rad51 (HR) was only recruited in G2 cells to sites of DSB induction (Figure S2B) [53].

As markers for the non-homologous end joining pathway we selected GFP-tagged versions of Ku70 as one of the early acting factors of NHEJ and DNA Ligase 4, which ligates the broken ends as a final step of this pathway (Figure 1C). mCherry-PCNA was again used as a reference for the induction of DNA lesions. A representative time-lapse series for the recruitment of GFP-Ligase 4 after micro-irradiation with the 405 nm laser is shown in Figure 1F. Quantitative analysis of the accumulation for GFP-Ligase 4 to sites of micro-irradiation clearly shows that the DNA DSB repair protein was only recruited to sites of 405 nm micro-irradiation but not after micro-irradiation with longer wavelengths (Figure 1G). A maximal accumulation of 1.5 fold was found for Ligase 4. (Figure 1H). Ku70-GFP accumulated also only at sites of 405 nm laser induced damage, but not after micro-irradiation with longer wavelengths. Here the maximum accumulation reached levels of approximately 1.3 fold (Figure 1H). We also checked for the cell cycle dependent accumulation and applied the same approach as described above. In contrast to Rad51, the Ligase 4 (NHEJ) was recruited to sites of laser micro-irradiation independent of the cell cycle stage (Figure S2C).

Altogether, this indicates that both DNA DSB pathways, homologous recombination and non-homologous end joining, get activated after micro-irradiation with 405 nm even in the absence of exogenous sensitizers, but not after micro-irradiation with longer wavelengths when low energy levels are used.

Addition of the sensitizers BrdU or EthBr did not lead to an activation of these pathways with longer wavelengths (shown for Ligase 4 and Ku70 in Table S1). Even after micro-irradiation at high energy levels (488 nm with 17 mJ), which resulted in the formation of γH2AX foci (Figure S1A), no accumulation of Ligase 4 nor Ku70 was detectable (Figure S2E). The later might also be due to the fact that high energies of 488 and 561 nm laser micro-irradiation irreversibly photo-bleaches the GFP labels of the tagged repair proteins. Nevertheless, NBS1, a subunit of the MRN complex (Figure 1C left) [54] did accumulate at sites of 488 nm induced DNA DSB (Figure S2E), when high energies were used. As γH2AX foci formation is a result of a local cellular amplification process with many neighbouring histones being phosphorylated resulting in a large focal accumulation of the factors involved, this likely explains why γH2AX can be detected under conditions when the other non-amplified repair factors are not detectable (reviewed in [55]).

Experiments with energy levels equivalent to our initial settings were also performed on a Leica SP5 II point scanning confocal microscope for Ligase 4 to test independency of the microscope and laser setup. Also in this point scanning confocal laser microscope the activation of DNA DSB repair pathway was exclusively seen after irradiation with 405 nm laser (Figure S2F).

Taken together, we could verify that DNA DSBs are induced after micro-irradiation with 405 nm CW lasers. Former studies routinely used additional sensitizers, mostly BrdU, to enhance the formation of DNA DSBs. We demonstrated that 1 mJ of a 405 nm CW laser is not only sufficient to induce DSBs without additional sensitizers, but also that the corresponding DNA repair pathways are activated. This allows the study of these DNA repair pathways in real time with high spatial resolution on conventional confocal microscopes without unwanted side effects from exogenous sensitizers. Furthermore the cell cycle stage of the micro-irradiated cells can be directly derived from the cell nucleus area, acquired during the micro-irradiation experiment.

### 3.3. Cyclobutane pyrimidine dimers are induced by 405 nm laser micro-irradiation

**Figure 2.**
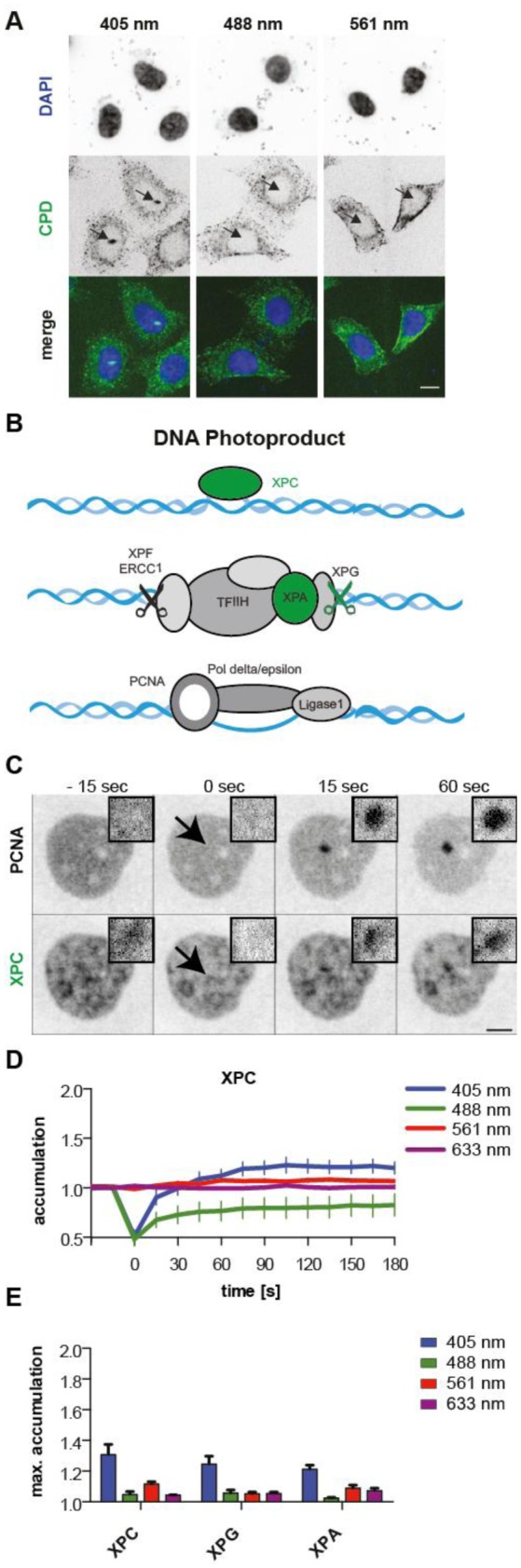
Micro-irradiation with 405 but not with 488 and 561 nm lasers induces DNA photo-products and activates nucleotide excision repair. (A) Immunofluorescent detection of cyclobutane dimers show a signal at the sites of micro-irradiation with 405 nm laser but no signal after 488 or 561 nm irradiation in HeLa cells. (B) Simplified schematics for the NER pathway with analyzed proteins highlighted in green. (C) HeLa Kyoto cells, stably expressing mCherry-PCNA transfected with XPC-GFP were micro-irradiated at the indicated spots and analyzed by time-lapse microscopy. Scale bar = 5 µm. (D) Measured mean accumulation of XPC-GFP after micro-irradiation with the indicated wavelength. Curves represent means and error bars are standard deviation. (E) Bar histograms display the calculated mean maximal accumulation for the different repair factors and wavelengths as indicated. Error bars represent standard deviation.

After the analysis of DSB repair pathways activated by micro-irradiation we analyzed the wavelength dependency of CPD induction and subsequent nucleotide excision repair (NER) activation. To directly assess the induction of pyrimidine dimers, we stained cells immediately after micro-irradiation with an anti-cyclobutane pyrimidine dimer (CPD) antibody. We detected CPDs exclusively after micro-irradiation with the 405 nm laser (1 mJ), but not after micro-irradiation with 488 or 561 nm lasers (3 and 8 mJ respectively, Figure 2A). Additionally, we tested if, at higher energy levels, CPDs could also be induced with the longer wavelengths. Indeed, CPD induction could be observed after irradiation with either 17 mJ 488 nm or 66 mJ 561 nm (Figure S3A). CPD induction is therefore largely dependent on the energy applied and differences between laser setups must be taken into account when comparing data sets from different studies. The mechanism of CPD induction at longer wavelength is not well understood, but the same mechanism as discussed above can be reasoned. Additionally, formation of CPDs can occur via the formation of an excited triplet state of the sensitizer. If the energy of the triplet is larger than that of thymidine in DNA, a transfer may take place and lead to the formation of CPDs. An alternative explanation would be a weak direct absorption of the DNA (reviewed in [56]), although unlikely at wavelengths of 488 and 561 nm.

### 3.4. Micro-irradiation with 405 nm lasers activates NER

Since we observed CPD formation after 405 nm micro-irradiation, we were interested if NER is activated. Therefore, we used XPC, one of the first factors in global genomic NER (Figure 2B) to study recruitment to damage sites. XPC can bind nonspecifically to undamaged DNA, but the binding is strongly enhanced at DNA with helix distortions where it initiates the NER [57]. XPC-GFP transfected cells therefore already showed structured fluorescent signal in the nucleus (Figure 2C). After micro-irradiation with the 405 nm laser, the NER factor got recruited to the site of damage. Micro-irradiation with longer wavelength on the other hand did not lead to an accumulation. Irradiation with the 488 nm laser even lead to a persistent lower fluorescence at the irradiated spot due to photobleaching of the unspecifically DNA-bound XPC-GFP fraction with the laser (Figure 2D). This reflects the low mobility (Figure S3B) of XPC that continuously probes DNA for damage and is in accordance to the data reported by [38]. The maximal accumulation for XPC-GFP after micro-irradiation with 405 nm laser reached an average of 1.3.

Since NER is dependent on p53 we confirmed the activation of NER also in p53 wild type human fibroblasts (GM00637; SV40 transformed skin fibroblast line). We could observe, at the same energy level, a higher accumulation (2.6) of XPC-GFP as compared to HeLa cells (Figure S3 C-E), but still no recruitment after 488 nm and 561 nm micro-irradiation using our standard irradiation settings.

Since it was shown that XPC binds to DNA helix distortions and needs subsequent verification of the damaged base before proceeding with NER and XPC was also shown to stimulate BER [15],[21],[38],[57], we validated the activation of NER with additional downstream NER factors. Although other studies have not found factors acting downstream of XPC to be recruited to 405 nm laser induced damage sites [16],[21], our conditions use more than a 10-fold higher irradiation exposure and do induce CPDs (Figure 2A). Thus, micro-irradiation experiments with fluorescently tagged XPG and XPA, which both act downstream of XPC, (Figure 2B), were performed. These NER factors also showed a specific recruitment to sites of 405 nm induced damage with a maximum accumulation of approximately 1.3 fold (Figure 2E). This relatively weak accumulation is in line with data reported by Kong et al. [14]. The recruitment after micro-irradiation with 405 nm laser of XPC and XPG were additionally validated in the confocal point scanning microscope (Figure S3F).

Addition of sensitizers (BrdU or EthBr) lead to an accumulation of XPC also when cells were irradiated with the 488 and 561 nm lasers although not as strong. Nevertheless, the accumulation of the downstream NER factor XPG was exclusively detected after micro-irradiation with the 405 nm laser (Table S1). Alternatively, XPC accumulation could also be found after 488 and 561 nm irradiation when higher energy levels were applied (17 and 66 mJ, respectively), in line with the observation that these energy levels induce CPDs (Figure S3A). Again the accumulation of the downstream NER factors could not be detected (Figure S3G). This reveals that full activation of the NER pathway is only achieved after micro-irradiation with the 405 nm laser. An accumulation of XPC without the recruitment of downstream NER factors was also reported by Menoni et al. [21] and Lan et al. [16], which is in line with the suggested additional role of XPC and CSB in the repair of oxidative DNA lesions.

So far studies on NER with micro-irradiation were mostly done with UVC lasers, which require special lenses and coupling the UVC laser into the microscope. We show here that it is also possible to activate NER using the 405 nm laser, attesting that it is also possible to study NER with conventional microscopes. However, the activation is weak, when low levels of laser energy are used.

### 3.5. Discrimination of non-processive against processive BER

**Figure 3.**
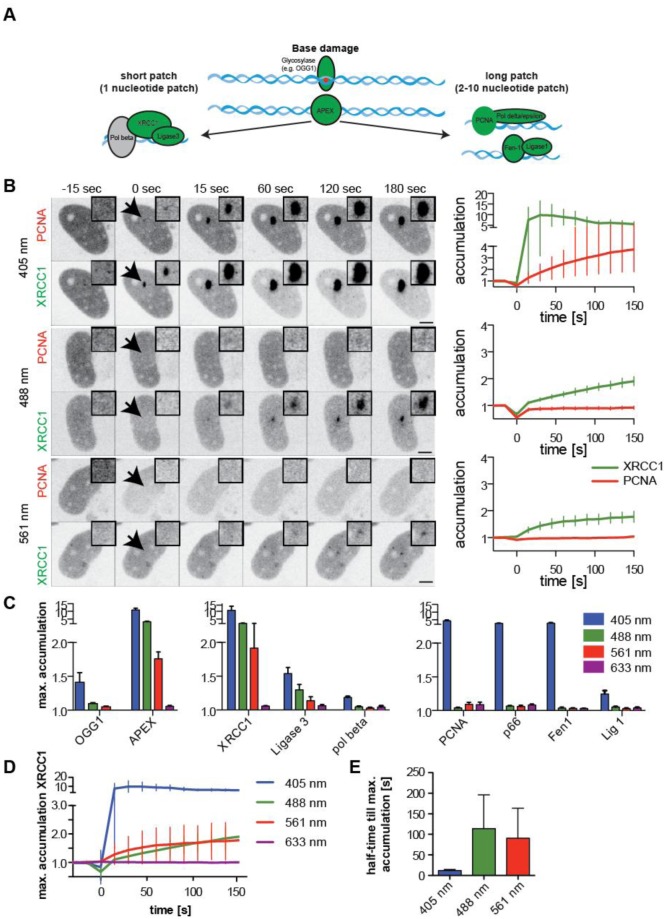
Discrimination against processive DNA repair with 405 versus 488 and 561 nm micro-irradiation. (A) Schematic representation of short- and long-patch base excision repair. (B) HeLa Kyoto cells stably expressing mCherry-PCNA were transfected with GFP-XRCC1 and irradiated with 405, 488 and 561 nm laser set to 1, 3, and 8 mJ respectively. Arrowheads point to the sites of micro-irradiation, shown enlarged as insets. Scale bars: 5 µm. The plots represent the accumulation of the different repair proteins over time as mean value. Error bars represent standard deviation. (C) Bar histograms display the calculated mean maximal accumulation for the different repair factors as indicated. Error bars represent standard deviation. (D) Different accumulation kinetics for XRCC1 after micro-irradiation with lasers of different wavelength- Lines present the mean values and error bars are calculated as standard deviations. (E) Half-times till maximum accumulation of XRCC1 after micro-irradiation with 405, 488 and 561 nm laser set to 1, 3, and 8 mJ respectively. Bars present means and error bars are standard deviations.

Since it is difficult to detect small amounts of damaged bases or abasic sites *in situ* and *in vivo*, we could not rule out the possibility that these kinds of damage are formed after micro-irradiation. In previous reports the recruitment of glycosylases OGG1, NEIL1, NEIL2, and NTH1 tagged with GFP were used to assess damaged bases and were shown to colocalize with 8-oxo-dG [16],[21]. These lesions are repaired by base excision repair (BER). BER can be divided into short- and long-patch BER (Figure 3A). During short-patch BER only one nucleotide is replaced. For this non-processive DNA synthesis, XRCC1 serves as a loading platform and Ligase 3 is used to seal the nick. XRCC1, though, has also been shown to play a role in the repair of SSBs [58] in a PARP1 dependent manner [17],[34]. In addition SSB repair can be initiated as an intermediate in regular BER where, depending on the chemical structure of the damage or the complexity of clustered damage, short patch BER is less efficient and long patch BER is activated instead [59],[60]. In long-patch BER, up to 10 nucleotides are synthesized. In this processive DNA synthesis PCNA is required as a loading platform, as it is in other processive DNA repair pathways like, e.g., NER. In this case the final ligation step is performed by DNA Ligase 1.

As BER is initialized by the DNA glycosylases, we analyzed the recruitment of OGG1 and APEX1 to sites of micro-irradiation. While APEX accumulated after 405, 488 and 561 nm laser micro-irradiation, OGG1 could only be detected with the 405 nm laser irradiation in a transient manner (Figure 3C). This is consistent with previous reports that also found the OGG1 accumulation to be weak and very transient [16].

To distinguish between processive and non-processive BER, we used GFP-tagged XRCC1, polβ and DNA Ligase 3 as markers for short-patch BER and mCherry-tagged PCNA, Ligase 1, Fen1 and polδ p66 as indicators for long-patch BER and other processive DNA repair pathways. Time lapse microscopy revealed a recruitment of all factors after micro-irradiation with 405 nm laser, with XRCC1 exhibiting a very strong accumulation (>10 fold) (Figure 3B and C). Irradiation with 488 or 561 nm lead to a significant accumulation of XRCC1 as well as Ligase 3 but not polβ, which also exhibited the lowest accumulation after 405 nm irradiation. Also 488 and 561nm laser micro-irradiation resulted in no accumulation of PCNA (Figure 3B and C), nor of any of the other long-patch-BER factors we tested (polδ p66, Fen1 and Ligase 1, Figure 3C). Notably, the accumulation of XRCC1 was slower after micro-irradiation with the longer wavelength compared to accumulation after 405 nm laser irradiation. Calculation of the half-time of accumulation after micro-irradiation revealed a prolonged half time to maximum accumulation from approximately 10 seconds for micro-irradiation with the 405 nm laser up to 100 seconds for 488 and 561 nm irradiation (Figure 3D and E).

These results indicate a preferred activation of short-patch-BER after irradiation of the cells with 488 or 561 nm lasers. Another possibility is the induction of single strand break (SSBs), which are also repaired in a XRCC1 and Ligase 3 dependent manner [61],[62]. The latter process is believed to be directly dependent on PARP1 as a recruitment factor for XRCC1 whereas the orchestrated BER process are thought to be less dependent on PARP when the base damage is channeled directly from glycosylases to ligases in a multi-protein complex [63]. This also explains why we observe different recruitment kinetics for XRCC1 accumulation at sites of laser micro-irradiation. While the fast accumulation following 405 nm irradiation is possibly caused by a mixture of SSBs and base damage induction and subsequent PARP activation with XRCC1 recruitment as well as PARP independent XRCC1 recruitment, the slower accumulation of XRCC1 seen after 488 and 561 nm irradiation might reflect XRCC1 recruitment in (short-patch) BER independent of PARP.

To rule out that accumulation only occurred when proteins are overexpressed, we tested the accumulation of endogenous XRCC1 and PCNA. After micro-irradiation cells were directly fixed and stained for the repair factors by immunofluorescence. For direct comparison, antibody staining against XRCC1 was performed in mCherry-PCNA expressing cells and PCNA antibody staining in RFP-XRCC1 expressing ones. Immunofluorescence staining for endogenous XRCC1 verified the accumulation of this short-patch BER factor to sites of 405, 488 and 561 nm induced damage. Accordingly, in RFP-XRCC1 transfected cells the fusion protein got recruited after micro-irradiation with 405, 488 and 561 nm laser, while accumulation of the endogenous PCNA was only observed after micro-irradiation with the 405 nm laser (Figure S4).

Irradiation with the 633 nm laser line never lead to the recruitment of any of the NER DNA repair factors tested, not even in the presence of exogenous sensitizers BrdU or EthBr (Figure 3C, 3D and Table S1). On the other hand sensitizations with EthBr, but not BrdU, lead to an accumulation of PCNA even to sites of 488 or 561 nm laser micro-irradiation. Nevertheless, this effect could not be observed for p66, which is another factor in processive DNA synthesis pathways (Table S1). The accumulation of PCNA was also detected when micro-irradiation with 488 or 561 nm was performed with high energies (17 mJ and 66 mJ respectively) in the absence of exogenous sensitizers (Figure S3G).

To rule out that the observed accumulations at sites of local micro-irradiation are setup specific we confirmed our results using another microscope type. We micro-irradiated fluorescently tagged XRCC1, PCNA or p66 expressing cells with 405, 488 and 561 nm laser at equal energy levels comparable to our initial settings in a confocal laser scanning microscope Leica SP5 II (point scanner). For XRCC1 we could validate the accumulation not only for 405 nm laser induced damage, but also recruitment after 488 and 561 nm laser micro-irradiation in HeLa cells (Figure S4B). The processive DNA synthesis related factors PCNA and p66 on the other hand, only accumulated at sites of 405 nm laser induced DNA damage. Also in this system the BER related factors accumulated at higher levels than factors specific for NER or DSB repair (Figure S4B compare to Figure 1 and 2).

### 3.6. Micro-irradiation with CW visible lasers in combination with PARP inhibition or methoxyamine treatment

**Figure 4.**
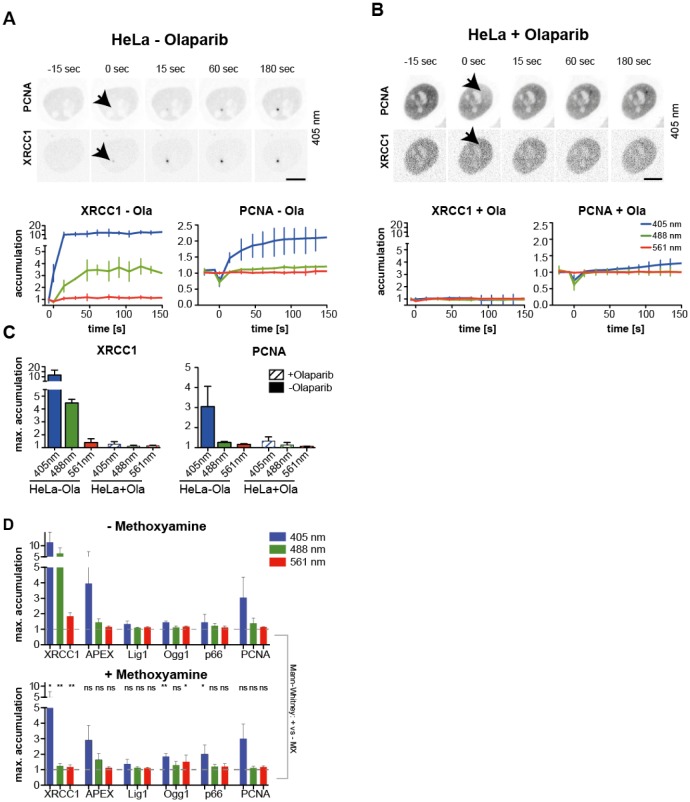
Micro-irradiation in PARP inhibited or methoxyamine treated cells. Time lapse imaging of HeLa Kyoto cells stably expressing mCherry-PCNA and transfected with GFP-XRCC1, either mock treated (A) or pretreated with Olaparib (B) and micro-irradiated with 405 nm. Lower panels shows the mean accumulation and error bars represent standard deviation. (C) Quantification of wavelength dependent accumulation of PCNA and XRCC1 in mock or Olaparib treated cells. Bars represent the mean maximum accumulation and error bars indicate the standard deviation. (D) Quantification of micro-irradiation induced maximum accumulation in cells not exposed (upper panel) or exposed to methoxyamine. Bars represent the means and error bars are standard deviation. The statistical significance of methoxyamine treatment was tested by a two-tailed Mann-Whitney-U test (95% confidence interval) between the treated and untreated sample and is indicated as *: *p* ≤ 0.05; **: *p* ≤ 0.01 and ns: *p* > 0.05.

To understand whether the XRCC1 accumulation after laser micro-irradiation is dependent on PARP, we studied XRCC1 accumulation in cells inhibited by Olaparib [59],[60],[64]. Olaparib is a PARP-1 and -2 inhibitor that prevents the formation of poly-ADP-ribose chains. Previously it was reported that PARP1 inhibition also prevents the accumulation of Fen1 [65] at sites of micro-irradiation, when a pulsed NIR laser is applied. When cells were pretreated with Olaparib XRCC1 accumulation was completely abolished (Figure 4A and B). Furthermore, we studied the wavelength dependent accumulation of XRCC1 in PARP1^−/−^ cells and compared it to the corresponding wild type cells. While wild type MEFs showed a XRCC1 recruitment comparable to HeLa cells after 405, 488 and 561 nm laser irradiation, the PARP^−/−^ cells showed a significantly reduced level of accumulation of XRCC1 (Figure S5A and B). Maximum accumulation in PARP^−/−^ cells was reduced from 18.0 ± 9.7 (wild type cells) to 2.4 ± 0.2 (mean + SE) after exposure to 405 nm laser. This suggests that the majority of damage induced by 405 nm laser irradiation are SSBs and only a minor fraction can be attributed to base damage (PARP1 independent). Also, we can conclude by comparing the Olaparib inhibited cells to the PARP1 knock out cells, that the recruitment of XRCC1 to SSBs is not only dependent on the PARP1 activity but to some extend also to PARP2 activity. Furthermore, the accumulation of PCNA after 405 nm exposure was reduced significantly in the Olaparib treated cells but to a lower extend in PARP1^−/−^ MEFs when compared to the untreated or wild type cells. This can be explained with a fraction of the damage being base damage and being repaired by the long-path BER or by the induction of different types of DNA damage that is repaired by, e.g., NER (see above). This is also reflected by the recruitment kinetics of PCNA to the sites of micro-irradiation, which shows a delayed recruitment in Olaparib treated or knock out cells.

Methoxyamine (MX), which reacts with the ribose moiety of an abasic site rendering it unsuitable for cleavage by APEX [66], was additionally used to further understand the XRCC1 recruitment. Therefore, we treated cells with MX and analysed the wavelength dependent accumulation of BER factors to discriminate between base damage and SSBs. While XRCC1 recruitment was significantly reduced after 405 nm (50%) and abolished at 488 and 561 nm irradiation, no inhibitory effect of MX treatment was measured for PCNA, DNA Ligase 1, p66, Ogg1 and APEX (Figures 4D and Figure S5D). The fact that APEX was still accumulated at sites of micro-irradiation can be explained by the fact that APEX1 directly interacts with XRCC1 [67] and/or that APEX is recruited but unable to process the MX-blocked AP sites. Furthermore, the MX inhibited cells showed an increased level of OGG1 as well as p66 after 405 nm micro-irradiation (Figure 4D). This is possibly due to the fact that the MX blocked site can still recruit the BER complex as discussed above.

Taken together the results from PARP inhibition suggest that the vast majority of lesions induced by 405, 488 and 561 nm micro-irradiation are SSBs or at least are repaired in a PARP dependent manner. The results from the MX treated cells suggest that with 405 nm laser irradiation, nonetheless, a substantial amount of base damage is also induced (the repair of which can be inhibited by MX) and that a fraction of 488 nm and 561 nm induced XRCC1 accumulation is also related to base damage, since the MX treatment nearly abolishes XRCC1 accumulation with longer wavelength irradiation. This hints to a novel potential role of XRCC1 and PARP in the processing of base damage.

## 4. Conclusions

In conclusion, we could show that it is possible to induce various types of DNA damage (e.g., DSB and CPDs) with visible light CW laser micro-irradiation. The activation of the corresponding DNA repair pathways NHEJ, HR and NER can be monitored when the 405 nm laser is applied at 1 mJ. This allows studying these pathways on multiple standard confocal microscopes, equipped with a variety of laser sources e.g., confocal spinning disk microscopes fitted with diode pumped solid state (DPSS) lasers as well as conventional point-scanning confocal microscopes equipped with both, DPSS or gas lasers (e.g., Argon ion). The activation of these pathways can be achieved even in the absence of exogenous sensitizers that could potentially result in unwanted side effects. When these standard settings are employed a high damage density is induced that causes clustered damages in close spatial proximity. This has to be considered if repair studies are performed and repair kinetics are compared to damage induced by other methods.

Additionally, we could demonstrate that it is possible to discriminate for non-processive short-patch BER and SSB repair over processive DNA repair pathways with longer wavelength lasers (488 nm) when the energy level is controlled and kept below 3 mJ, respectively.

Finally, our studies underscore the importance of the energy and wavelength applied in a micro-irradiation setup for the induction of DNA damage and repair, if quantitative conclusions are drawn or different studies are compared (see also [6]).

Notably, the maximal accumulation of BER factors after micro-irradiation with 405 nm lasers is higher and faster than that for DSB repair or NER specific factors. This strengthens the model that the predominant DNA damage produced by low power visible CW lasers arises from absorption by endogenous cellular photosensitizers leading mainly to oxidative base damage and SSBs, which are in turn processed by the BER/SSB pathway.
