# Supplementary material for: Systematic analysis of DNA damage induction and DNA repair pathway activation by continuous wave visible light laser micro-irradiation
Source: AIMS Genet. 2017 Feb 21;4(1):47–68. doi: 10.3934/genet.2017.1.47 (PMC6690239; doi:10.3934/genet.2017.1.47)
Supplement: Supplementary file 1 [file genetics-04-01-047-s01.pdf]

AIMS Genetics

AIMS Genetics, 4(1): 47-68.
DOI: 10.3934/genet.2017.1.47
Received: 15 December 2016
Accepted: 16 February 2017
Published: 21 February 2017

http://www.aimspress.com/journal/Genetics

Research article

Systematic analysis of DNA damage induction and DNA repair pathway activation by continuous wave visible light laser micro-irradiation

Running title: Discrimination of DNA damage repair

Britta Muster†, Alexander Rapp† and M. Cristina Cardoso*

Cell Biology and Epigenetics, Department of Biology, Technische Universität Darmstadt, 64287 Darmstadt, Germany

* **Correspondence:** Email: cardoso@bio.tu-darmstadt.de; Tel: +49-6151-16-21882; Fax: +49-6151-16-21880.

† These authors contributed equally to this work.

Abstract: Laser micro-irradiation can be used to induce DNA damage with high spatial and temporal resolution, representing a powerful tool to analyze DNA repair *in vivo* in the context of chromatin. However, most lasers induce a mixture of DNA damage leading to the activation of multiple DNA repair pathways and making it impossible to study individual repair processes. Hence, we aimed to establish and validate micro-irradiation conditions together with inhibition of several key proteins to discriminate different types of DNA damage and repair pathways using lasers commonly available in confocal microscopes. Using time-lapse analysis of cells expressing fluorescently tagged repair proteins and also validation of the DNA damage generated by micro-irradiation using several key damage markers, we show that irradiation with a 405 nm continuous wave laser lead to the activation of all repair pathways even in the absence of exogenous sensitization. In contrast, we found that irradiation with 488 nm laser lead to the selective activation of non-processive short-patch base excision and single strand break repair, which were further validated by PARP inhibition and metoxyamine treatment. We conclude that these low energy conditions discriminated against processive long-patch base excision repair, nucleotide excision repair as well as double strand break repair pathways.

Keywords: DNA repair; DNA damage; processive DNA synthesis; laser micro-irradiation; live-cell microscopy

Supplementary material

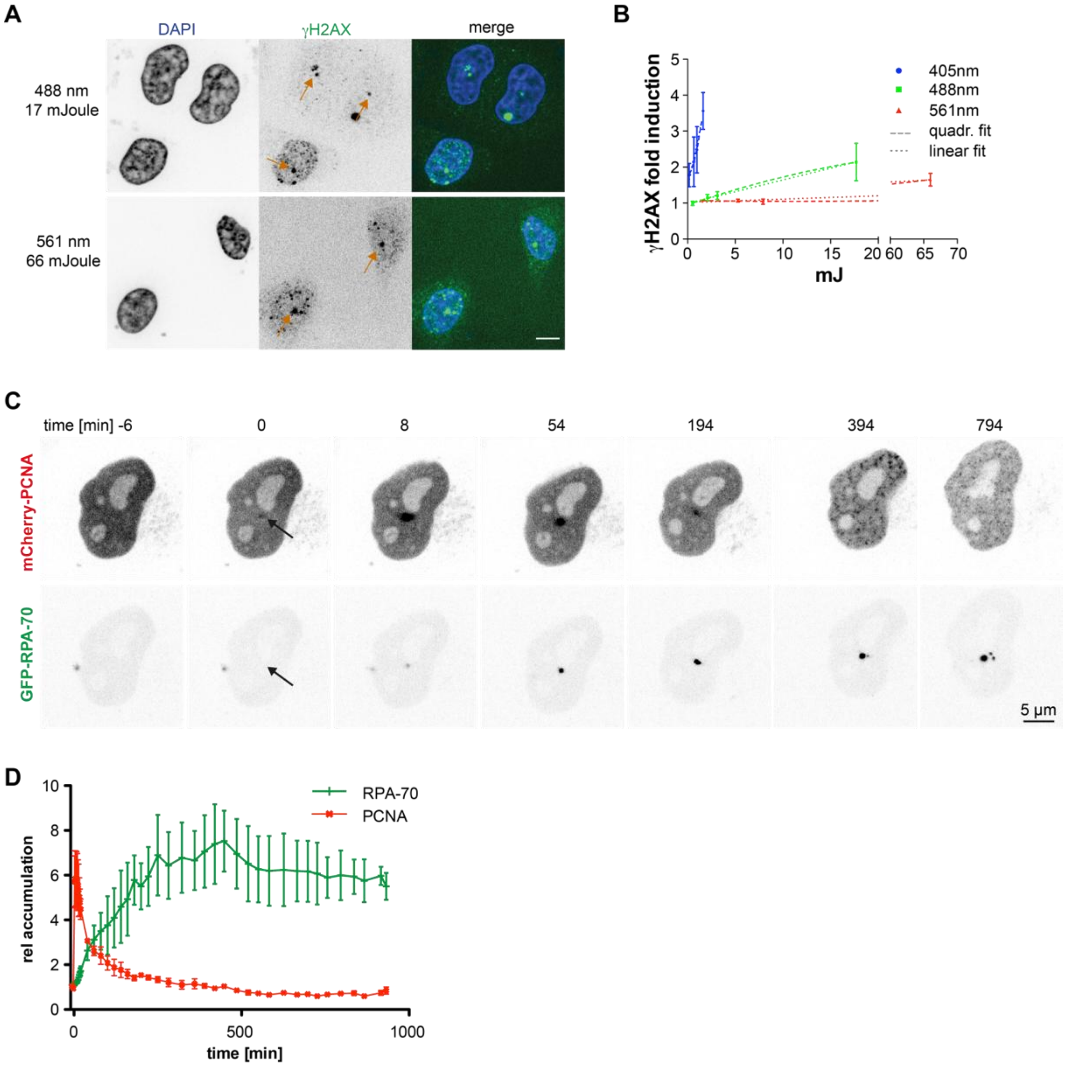

Figure S1. (A) HeLa cells were irradiated with high laser energies of 488 and 561 nm respectively. γH2AX focus formation was visualized by immunofluorescence. Scale bar: 5 µm. (**B)** Wavelength and energy dependent formation of γH2AX foci. The induction of γH2AX following micro-irradiation with different wavelength and energy settings were analyzed by measuring the intensity of the immunostained γH2AX in relation to the total nuclear background. The fold change of induction was calculated as the ratio between mean intensity at the site of irradiation to the mean nuclear intensity. The datasets were fitted with a linear (dotted) and a quadratic function (dashed). (**C)** Formation and resolution of 405 nm (1 mJ) laser induced foci of mCherry-PCNA and GFP-RPA-70. Cells were imaged for 16 hours, and images are shown with the indicated times in minutes. Scale bar 5 µm. (**D)** Quantification of the accumulation and release in C). Plotted are mean values and standard deviation.

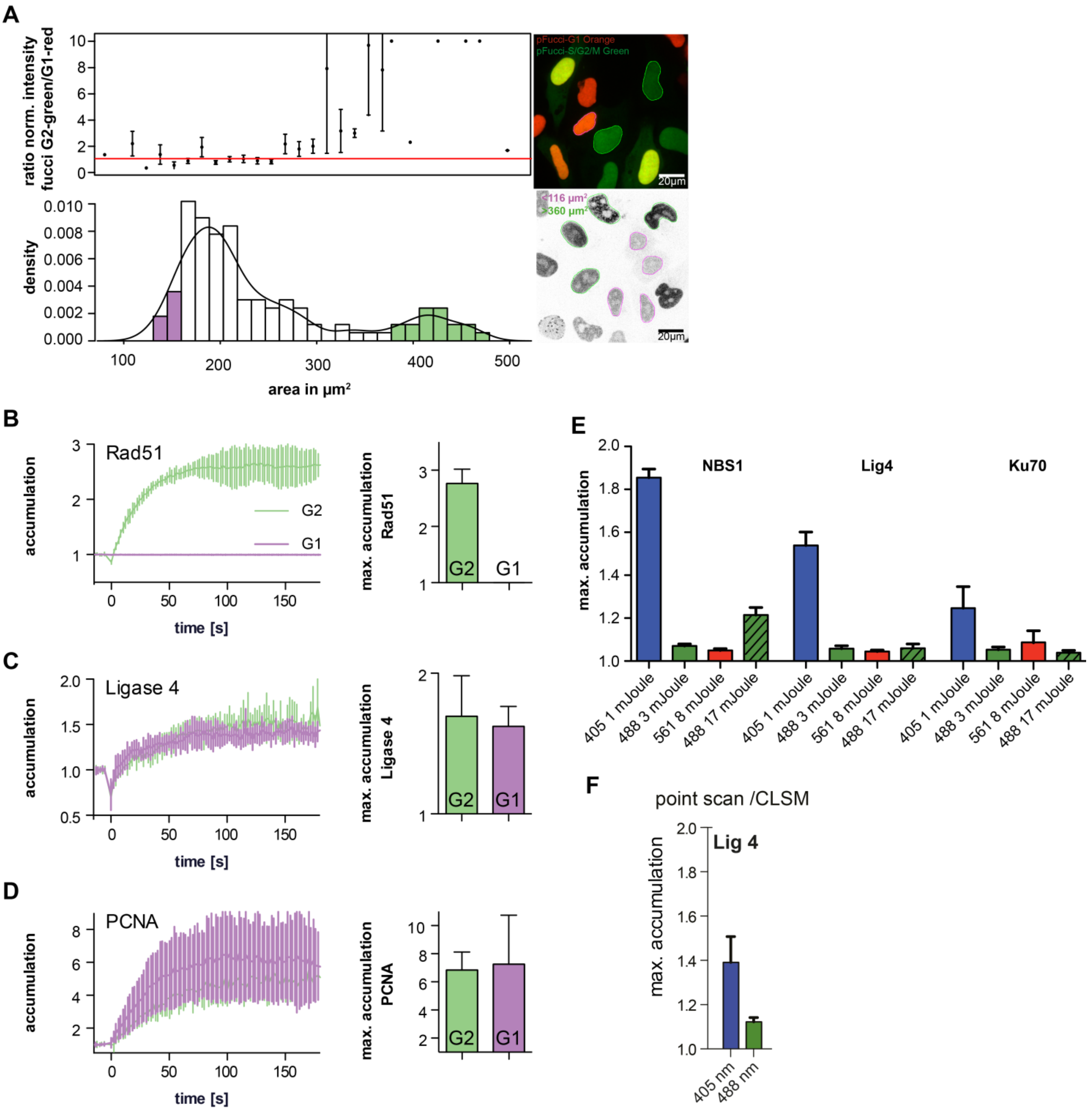

Figure S2. A) Correlation between the Fucci-green to Fucci-red ratio to the nucleus area of HeLa cells used in micro-irradiation. The image shows a merge of the two Fucci markers, scale bar 20 µm. The purple and green bars show the cells used to segment for G1 versus G2 cells and a sample image is shown on the right with DAPI staining; scale bar: 20 µm. **B)** Accumulation kinetics of Rad51 to sites of 405 nm radiation separated in G2 and G1 cells with the corresponding maximum accumulation bar plots. The line and bars represent means the error bars indicate standard deviation. **C and D)** Same as in B but for the accumulation of Ligase 4 and PCNA respectively. **E)** Maximum accumulation of DSB repair proteins after different laser energies and wavelength as indicated. Bars represent the mean and whiskers denote the standard deviation. **F)** Maximum accumulation of Ligase 4 after 405 and 488 nm micro-irradiation using a point scanning confocal system with similar energy settings for micro-irradiation. Again mean and standard deviation are plotted.

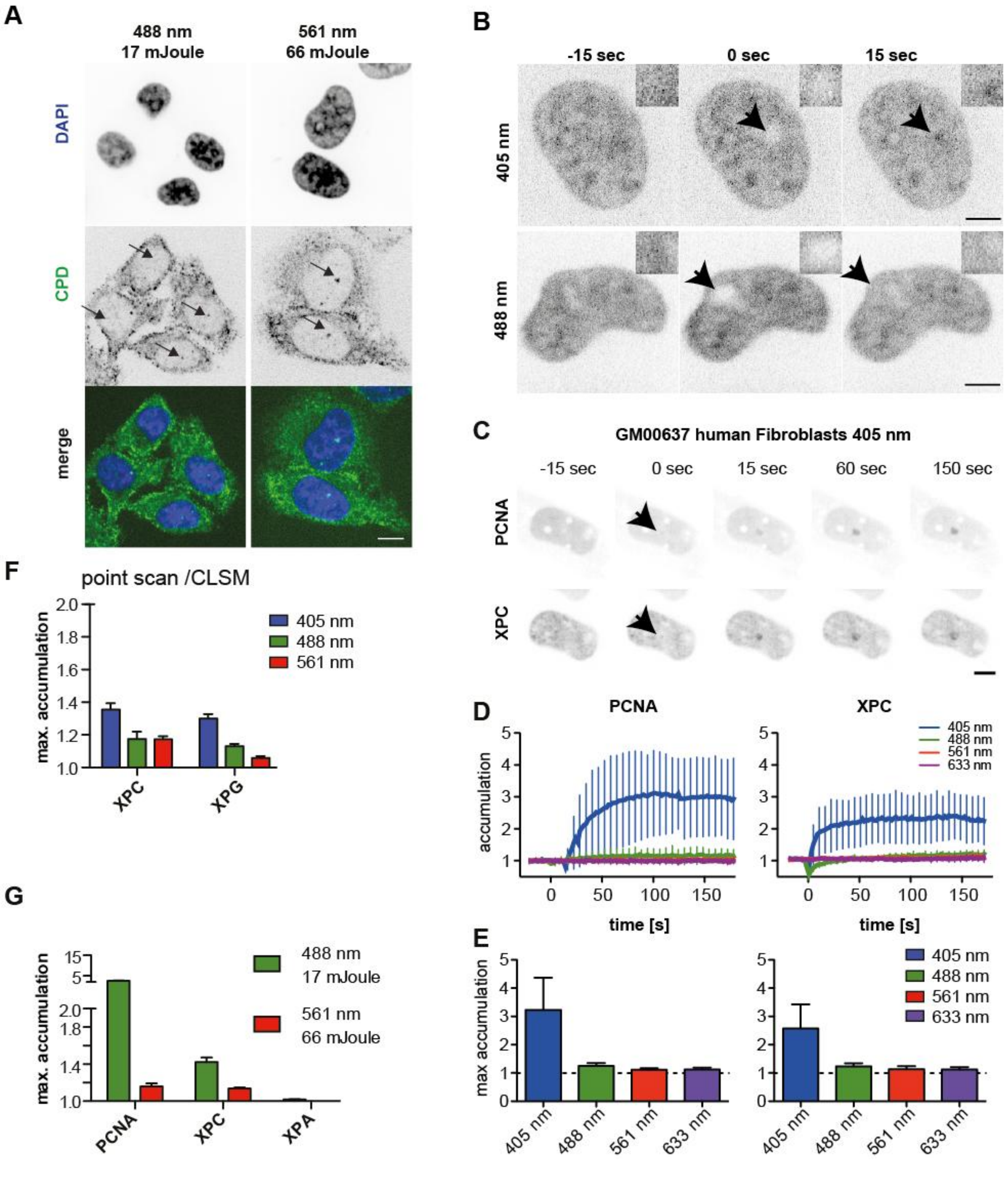

Figure S3. A) Induction of CPDs by higher energy levels in HeLa cells. Sites of micro-irradiation are denoted by the arrow. Scale bar: 5 µm. **B)** XPC-GFP transfected HeLa cells after micro-irradiation with 405 and 488 nm shown in time lapse microscopy. The arrows indicate the sites of micro-irradiation, scale bar: 5 µm. **C)** Wild-type human fibroblasts transfected with mCherry-PCNA and XPC-GFP, arrows indicate the location of the micro-irradiation; scale bar: 5 µm. **D)** Mean accumulation and standard deviation of XPC and PCNA accumulation in wild type fibroblasts. **E)** Mean maximum accumulation of the data shown in D, whiskers represent the standard deviation. **F)** Reproduction of the mean maximum recruitment and standard deviation of XPC and XPG using a point scanning confocal. **G)** Mean maximum accumulation and standard deviation for the accumulation of PCNA, XPC and XPA using higher laser energies as indicated.

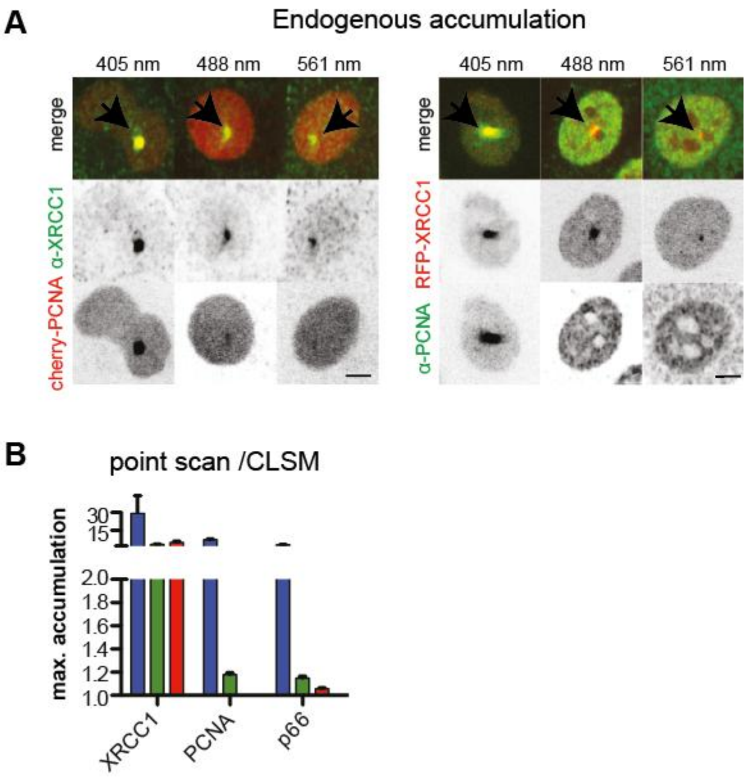

Figure S4. A) Accumulation of endogenous XRCC1 and PCNA in micro-irradiated HeLa cells. Endogenous proteins were co-stained with GFP/mCherry–fusions as indicated and fixed directly after irradiation. Scale bar: 5 µm. **B)** Average maximum accumulation of selected BER factors irradiated and imaged with a point scanning confocal microscope (CLSM). Whiskers indicate the standard deviation.

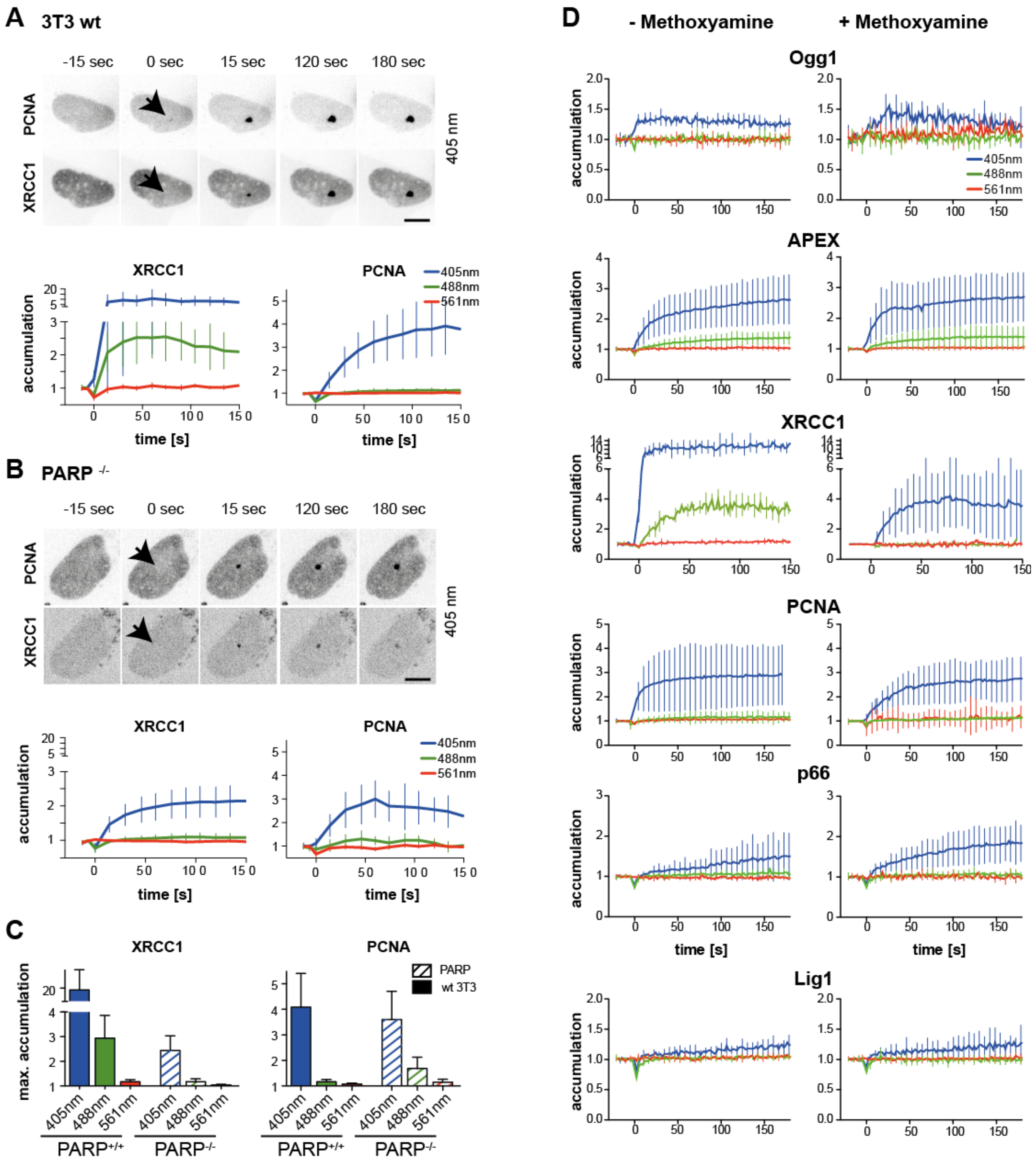

Figure S5. A) XRCC1 and PCNA accumulation at sites of 405 nm micro-irradiation extracted from time lapse microscopy in 3T3 wild type cells. Arrows indicate the sites of micro-irradiation, scale bar: 5 µm. Quantification of the accumulation depending on the wavelength is shown in the panels below. The lines represent means and the error bars are the standard deviation**. B)** Same as in A, but PARP$^{-/-}$ MEFs were used. **C)** The mean maximum accumulation for the wild type 3T3 (PARP$^{+/+}$) as well as for the PARP$^{-/-}$ are shown. Whiskers indicate the standard deviation. **D)** Wavelength dependent accumulation of BER factors in HeLa cells pretreated or mock treated with methoxyamine. Lines represent means and whiskers indicate the standard deviation.

Table S1. Summary of repair factor accumulation at micro-irradiation sites. Mean maximal accumulation after micro-irradiation from different fluorescently tagged DNA repair factors from at least three experiments. Experiments were performed either with BrdU, EthBr or without any exogenous sensitizers. Indicated lasers were used at specified bleaching times, resulting in 0.15, 0.6 and 1 mJ (405 nm), 0.5, 2 and 3 mJ (488 nm), 1.3, 5.2 and 8 mJ (561 nm) and 0.8, 3.2 and 5 mJ for the 633 nm laser line. Color-coding illustrates the level of accumulation ranging from orange representing no accumulation, over yellow for a mild accumulation of ≥ 1.2 fold and green for a strong accumulation greater or equal ≥ 2 fold.

max. accumulation	without sensitizer			BrdU			EthBr		
**Ku70 (NHEJ DSB)**	200 ms	800 ms	1200 ms	200 ms	800 ms	1200 ms	200 ms	800 ms	1200 ms
405	1.1	1.1	1.2		1.2	1.3			1.1
488			1.0			1.0			1.1
561			1.1			1.1			1.1
633			1.0			1.1			
**Ligase 4 (NHEJ DSB)**									
405	1.2	1.3	1.5			1.5	1.1	1.3	1.4
488			1.0			1.1			1.1
561			1.0			1.1			1.2
633			1.0			1.1			1.1
**Rad51 (HR DSB)**									
405			1.5			2.2			3.6
488			1.1			1.1			1.2
561			1.1			1.1			1.2
633									
**XPC (NER)**									
405	1.2	1.4	1.3	3.5		3.0		1.3	2.0
488			1.0			1.2	1.2	1.1	1.3
561		1.1	1.1			1.2			1.7
633	1.0		1.0			1.1			1.1
**XPG (NER)**									
405			1.2			1.4			1.1
488			1.0			1.0			1.0
561			1.0			1.0			1.1
633			1.0			1.0			1.0
**p66 (BER+NER)**									
405	1.6	1.5	3.0	1.9	2.3	2.3	1.6	2.1	2.1
488			1.1			1.0			1.1
561			1.1			1.1			1.1
633			1.1			1.0			1.1
**PCNA (BER+NER)**									
405	2.1	4.2	5.4	5.4	8.1	9.2	2.9	5.2	6.2
488	1.1	1.2	1.1	1.2		1.1	1.1		1.7
561	1.0	1.1	1.1	1.1	1.3	1.1	1.3		2.3
633			1.1			1.0			1.1
**XRCC1 (BER)**									
405	7.3	12.1	10.6	15.7	7.3	12.8	16.3		10.1
488	1.3	3.3	2.2	3.5	5.9	8.5	2.2		6.1
561	1.2	1.9	1.9	2.6	4.6	3.6	3.8		9.9
633			1.0			1.1			1.1
	≤1.1	1.2-1.9	≥2						
